# Vapor Pressure Formulation for Ice

**DOI:** 10.6028/jres.081A.003

**Published:** 1977-02-01

**Authors:** Arnold Wexler

**Affiliations:** Institute for Basic Standards, National Bureau of Standards, Washignton, D.C. 20234

**Keywords:** Clausius-Clapeyron equation, saturation vapor pressure over ice, thermal properties of ice, vapor pressure, vapor pressure at the triple point, vapor pressure of ice, water vapor

## Abstract

A new formulation is presented for the vapor pressure of ice from the triple point to −100 °C based on thermodynamic calculations. Use is made of the definitive experimental value of the vapor pressure of water at its triple point recently obtained by Guildner, Johnson, and Jones. A table is given of the vapor pressure as a function of temperature at 0.1-degree intervals over the range 0 to −100 °C, together with the values of the temperature derivative at 1-degree intervals. The formulation is compared with published experimental measurements and vapor pressure equations. It is estimated that this formulation predicts the vapor pressure of ice with an overall uncertainty that varies from 0.016 percent at the triple point to 0.50 percent at −100 °C.

## 1. Introduction

In meteorology, air conditioning, and hygrometry, particularly in the maintenance and use of standards and generators in calibrations and in precision measurements, accurate values of the vapor pressure of the pure water-substance are essential. Because of this Wexler and Greenspan [1]^1^ recently published a new vapor pressure formulation for the pure liquid phase, based on thermodynamic calculations, which is in excellent agreement from 25 to 100 °C with the precise measurements of Stimson [2]. Wexler [3] subsequently revised this formulation to make it consistent with the definitive experimental value of the vapor pressure of water at its triple point obtained by Guildner, Johnson, and Jones. The purpose of this present paper is to apply a similar method of calculation to the pure ice phase and derive a new formulation for temperatures down to −100 °C. This new formulation for ice is constrained to yield the identical value of vapor pressure at the triple point as that given by the revised formulation for the liquid phase.

A critical examination of the experimental vapor-pressure data of ice discloses the disconcerting fact that the dispersion among those values far exceeds modern accuracy requirements. This dispersion arises, in part, from the inherent difficulties experienced by investigators in making precision measurements of these low pressures and from the ambiguities in the temperature scale used in the early 1900’s when several major series of determinations were made. Thermodynamic calculations, based on accurate thermal data, provide an alternate route to the determination of vapor pressure. It is therefore not surprising that such calculations have been made repeatedly for ice with varying degrees of success. It is interesting to note that these calculations have been preferred over the existent experimental vapor pressures, primarily because the calculations appear to yield less uncertainty than the measurements.

## 2. Derivation

The Clausius-Clapeyron equation, when applied to the solid-vapor phase transition for the pure water-substance, may be written

$$\frac{dp}{dT}=\frac{l}{T(v-v^{\prime \prime })}$$ (1)

where *p* is the pressure of the saturated vapor, *v* is the specific volume of the saturated vapor, *v*″ is the specific volume of the saturated ice, *T* is the absolute thermodynamic temperature, *l* is the latent heat of sublimation, and *dp*/*dT* is the derivative of the vapor pressure with respect to temperature. The latent heat of sublimation is given by

$$l=h-h^{\prime \prime }$$ (2)

where *h* is the specific enthalpy of saturated water vapor at temperature *T* and *h*″ is the specific enthalpy of saturated ice at the same temperature *T.*

The equation of state for saturated water vapor may be expressed by

pv=ZRT (3)

where *Z* is the compressibility factor and *R* is the specific gas constant. When eq (3) is substituted into eq (1) the result is

$$\frac{dp}{p}=\frac{l}{ZRT^{2}}\left(1+\frac{v^{\prime \prime }}{v}\right)dT$$ (4)

where higher order terms of *v″*/*v* are neglected because *v″*/*v <<* 1. On integrating, eq (4) becomes

$$\int_{p_{1}}^{p_{2}}d(\ln\;p)=\int_{T_{1}}^{T_{2}}\frac{l}{ZRT^{2}}\left(1+\frac{v^{\prime \prime }}{v}\right)dT$$ (5)

where *p*_1_ and *p*_2_ are the saturation vapor pressures at temperatures *T*_1_ and *T*_2_, respectively. Suitable functions will be sought for Z, *v, v"* and *l* in order to complete the integration of eq (5).

Functions for the compressibility factor *Z* and the specific volume of saturated water vapor *v* will be based on a virial equation of state expressed as a power series in *p*. A function for the specific volume of saturated ice *v*″ will be developed from experimental data for the coefficient of linear expansion and the density at 0 °C. A function for the latent heat of sublimation *l* will be derived from the specific enthalpies *h*″ and *h* of saturated ice and saturated water vapor, respectively. Use will be made of measurements of the specific heat of ice to obtain *h*″ whereas statistical mechanical calculations of the ideal-gas (zero-pressure) specific heat of water will serve as input data for establishing an expression for *h.*

### 2.1 Temperature

Guildner and Edsinger [5] have recently made measurements on the realization of the thermodynamic temperature scale from 273.16 to 730 K by means of gas thermometry. Unfortunately there are no similar high precision measurements below 273.16 K. Therefore, it will be assumed that the International Practical Temperature Scale of 1968 (IPTS-68) [6] is a sufficiently close approximation to the absolute thermodynamic temperature so that the thermal quantities given in terms of IPTS-68 can be used in eq (5). From the triple point to −100 °C the temperature *t* in degrees Celsius has the same numerical values on the International Temperature Scale of 1927 (ITS-27) [7], the International Temperature Scale of 1948 (ITS-48) [8] and the International Practical Temperature Scale of 1948 (IPTS-48) [9]. However, the ice point on IPTS-48 is defined as equal to 273.15 kelvins whereas on ITS-27 and ITS-48 it is defined as equal to 273.16 kelvins. The difference *T*_68_ – *T*_48_, where *T*_68_ and *T*_48_ are the kelvin temperatures on IPTS-68 and IPTS-48, respectively, in the range of interest reaches a maximum of 0.0336 kelvin at 200 K [10, 11]. Using the corrections given by Riddle, Furukawa, and Plumb [11], temperatures on ITS-27, ITS-48 and IPTS-48 have been converted to IPTS-68 where needed in the calculations.

### 2.2. Specific Volume of Saturated Vapor

Equation (3) is used to calculate the specific volume of saturated water vapor *v.* The compressibility factor *Z* is expressed as a power series in *p*

$$Z=1+B^{\prime }p+C^{\prime }p^{2}+\cdots$$ (6)

where *B*′ is the second pressure-series virial coefficient and *C*′ is the third pressure-series virial coefficient. The contribution of *C*′ to *Z* is only a few parts per million at the triple point and less at lower temperatures and so has negligible effect. The empirical relationship for the second virial coefficient is based on experimental data obtained at elevated temperatures. This equation will be extrapolated below 0 °C with the full recognition that this may lead to large uncertainties in the virial coefficients. Although *B*′ rapidly increases in magnitude with decreasing temperature, the saturation vapor pressure decreases even more rapidly so that *Z* rapidly approaches its limiting value of unity as the temperature drops. Saturated water vapor, therefore, tends to behave more and more like an ideal gas as the temperature decreases, thereby reducing the effect of errors in *B*′.

Table 1 shows a comparison of *Z* from the triple point to −100 °C, for water vapor saturated with respect to ice, calculated using the empirical second virial coefficient equations of Goff and Gratch [12, 13, 14], Keyes [15, 16], and Juza as given by Bain [17]. The maximum difference in Z, as well as *v* is 118 ppm and occurs at 0.01 °C. This can be used as an indication of uncertainty although the actual error is indeterminate. The differences decrease as the temperature decreases. At −70 °C and below, the differences are equal to, or less than, one ppm since, at such temperatures, the second virial coefficient makes a negligible contribution to *Z*.

The 1969 second virial coefficient of Keyes [16] will be used in order to be consistent with the earlier use of this same coefficient [3]. His virial coefficient equation, converted to SI units compatible with eq (6), is

$$B^{\prime }=\left[\frac{0.44687}{T}-\left(\frac{565.965}{T^{2}}\right)\times 10^{\frac{100800}{34900+T^{2}}}\right]\times 10^{-5}.$$ (7)

where *B*′ is in units of reciprocal pressure (Pa)^−1^.^2^

### 2.3. Specific Volume of Saturated Ice

Only hexagonal Ice-I will be of concern. It will be assumed that the crystals are randomly aligned with respect to the optic axis. All known measurements of the density of ice have been made in the presence of an inert gas, usually at a pressure of one atmosphere and at a temperature of 0 °C. Dorsey [18] has compiled an extensive list of such determinations. Ginnings and Corruccini [19] using a Bunsen ice calorimeter, obtained a value at 0 °C and one atmosphere^3^ of 0.91671 g/ml. This value is definitive and supersedes all earlier measurements. Using this value and the coefficient of linear expansion of ice, the specific volume was calculated at temperatures below 0 °C as follows.

The isopiestic coefficient of linear expansion of ice *α_P_* is defined by the equation

$$\alpha_{P}={\left(\frac{1}{\lambda_{i}}\frac{d\lambda }{dT}\right)}_{P}$$ (8)

where λ*_i_* is the initial length of a specimen at the ice point temperature, λ is the length of the same specimen at temperature *T* and *d*λ*/dT* is the rate of thermal expansion. By integrating eq (8), cubing the resultant equation, neglecting higher order terms in $\int_{T_{i}}^{T}\alpha_{P}dT$, it follows that

$${v^{\prime \prime }}_{P,\;T}={v^{\prime \prime }}_{P,\;T_{i}}\;\left[1+3\int_{T_{i}}^{T}\alpha_{P}\;dT\right]$$ (9)

where *v″_P,T_* is the specific volume of ice at pressure *P* and temperature *T* ${v^{\prime \prime }}_{P,T_{i}}$ is the specific volume of ice at pressure *P* and at the ice point temperature *T_i_.*

There are several series of measurements of the coefficient of linear expansion of hexagonal Ice-I at atmospheric pressure. The data of Jakob and Erk [20], Powell [21], Butkovich [22], Dantl [23], and LaPlaca and Post [24] were fitted to a linear equation by the method of least squares. The result is

$$\alpha_{P_{a}}\times 10^{6}=-7.6370+0.227097\;T$$ (10)

which, when substituted into eq (9) together with the Ginnings and Corruccini value^4^ for the density of ice at 0 °C and one atmosphere becomes

$$\begin{array}{r} {v^{\prime \prime }}_{P_{a},T}=1.069989-0.249933\times 10^{-4}T\\ +0.371606\times 10^{-6}T^{2} \end{array}$$ (11)

where ${v^{\prime \prime }}_{P_{a},T}$ expressed in cm^3^/g, is the specific volume at atmospheric pressure, i.e., 101325 Pa, and temperature *T.* It is the specific volume at saturation rather than at atmospheric pressure that is needed. The specific volume at a given pressure can be corrected to that at another pressure from a knowledge of the isothermal compressibility *k*, which is given by the equation

$$k=k_{s}+\frac{Tv^{\prime \prime }\beta^{2}}{{c^{\prime \prime }}_{P}}$$ (12)

where *k_s_* is the adiabatic compressibility, *T* is the absolute temperature, v″ is the specific volume, *β* is the volume expansivity, and *c″p* is the specific heat at constant pressure. Values of the isothermal compressibility of ice were calculated by using Leadbetter’s values [27] for the adiabatic compressibility of Ice-I, eq (11) to obtain the specific volume at pressure *P_a_* and temperature *T*, eq (10) to obtain $\beta (=3\alpha_{P_{a}})$, and eq (19) (which is derived later) to obtain the specific heat at constant pressure *P_a_*. The results for the temperature range of interest are given by the linear equation

$$k=(8.875+0.0165T)\times 10^{-11}$$ (13)

where *k* is expressed in units of (Pa)^−1^. The specific volume of ice at pressure *P* and temperature *T* is therefore ${v^{\prime \prime }}_{P,T}={v^{\prime \prime }}_{P_{a},T}[1-k(P-P_{a})]$ so that

$${v^{\prime \prime }}_{P,T}={v^{\prime \prime }}_{P_{a},T}[1-(8.875+0.0165T)(P-101325)\times 10^{-11}]$$ (14)

where *P* is expressed in Pa. If the saturated vapor pressure *p* is inserted for *P*, then eq (14) yields the pure phase specific volume *v*″ at saturation. Over the temperature range 173.15 to 273.16 K the numerical value of the bracket is equal to 1.000013 with a maximum variability of one ppm. Using this value yields

$$\begin{array}{r} {v^{\prime \prime }}_{P,T}=v^{\prime \prime }=1.070003-0.249936\times 10^{-4}T\\ +0.371611\times 10^{-6}T^{2}. \end{array}$$ (15)

### 2.4 Enthalpy of Ice

It can be shown [26] that the specific enthalpy *h*″ of the solid phase of a pure substance, say ice, is given by the relationship

$$dh^{\prime \prime }={c^{\prime \prime }}_{P}\;dT+{v^{\prime \prime }}_{T}\;dP-T{\left(\frac{\partial {v^{\prime \prime }}_{T}}{\partial T}\right)}_{P}dP$$ (16)

where *c″_P_* is the specific heat of ice at constant pressure *P.* When integrated this equation becomes

$$\int_{{h^{\prime \prime }}_{1}}^{{h^{\prime \prime }}_{2}}dh^{\prime \prime }=\int_{T_{1}}^{T_{2}}{c^{\prime \prime }}_{P}\;dT+\int_{P_{1}}^{P_{2}}{\left[v^{\prime \prime }-T{\left(\frac{\partial v^{\prime \prime }}{\partial T}\right)}_{P}\right]}_{T}dP$$ (17)

Because eq (17) represents a system undergoing a reversible process between two equilibrium states, the initial and final enthalpies are independent of the path. Therefore, a path is chosen which starts on the saturation curve at (*T_i_*, *p_i_*), moves isothermally to (*T_i_*, *P_a_*), then proceeds isobarically to (*T, P_a_*), and finally goes isothermally to (*T*, *p*). The integration along this path is given by

$$\begin{array}{l} h^{\prime \prime }-{h^{\prime \prime }}_{i}=\int_{p_{i}}^{P_{a}}{\left[v^{\prime \prime }-T{\left(\frac{\partial v^{\prime \prime }}{\partial T}\right)}_{p_{i}}\right]}_{T_{i}}dP\\ +\int_{p_{i}}^{T}{c^{\prime \prime }}_{P_{a}}\;dT+\int_{P_{a}}^{p}{\left[v^{\prime \prime }-T{\left(\frac{\partial v^{\prime \prime }}{\partial T}\right)}_{P_{a}}\right]}_{T}dP. \end{array}$$ (18)

if *p_i_* is the saturation vapor pressure at the ice-point temperature *T_i_, p* is the saturation vapor pressure at temperature *T* and *P_a_* is any other pressure, say standard atmospheric pressure, then *h″ − h″_i_* is the difference in specific enthalpy of saturated ice, under its own equilibrium vapor pressure, between temperatures *T* and *T_i_.*

Although measurements of the isopiestic specific heat of ice have been made by several investigators [28–35], only those of Giauque and Stout [34] will be used because it is believed that these are the best available over the range of temperatures of interest here. These measurements were made at standard atmospheric pressure and cover the temperature range 16.43 to 267.77 K. They are in good agreement with the precise measurements of Dickinson and Osborne [30]. Unfortunately, the latter measurements do not extend below 233.15 K.

Fitting the Giauque and Stout data from 169.42 to 267.77 K to a quadratic equation by the method of least squares with the temperature converted to IPTS-68 and the heat units to joules, yields

$${c^{\prime \prime }}_{P_{a}}=A_{0}+A_{1}T+A_{2}T^{2}$$ (19)

where ${c^{\prime \prime }}_{P_{a}}$ is the specific heat in J/gK at a pressure of one atmosphere. The coefficients are given in table 2. Integrating eq (19), one obtains

$$\begin{array}{l} \overset{T}{\underset{T_{i}}{\int }}{c^{\prime \prime }}_{P_{a}}\;dT=A_{0}(T-T_{i})\\ \;+\frac{A_{1}}{2}(T^{2}-T_{i}^{2})+\frac{A_{2}}{3}(T^{3}-T_{i}{\;}^{3}). \end{array}$$ (20)

By letting

$$\begin{array}{l} \Delta h^{\prime \prime }=\int_{p_{i}}^{P_{a}}{\left[v^{\prime \prime }-T{\left(\frac{\partial v^{\prime \prime }}{\partial T}\right)}_{p_{i}}\right]}_{T_{i}}dP\\ \;+\int_{p_{a}}^{P}{\left[v^{\prime \prime }-T{\left(\frac{\partial v^{\prime \prime }}{\partial T}\right)}_{p_{a}}\right]}_{T}dP \end{array}$$ (21)

and performing the indicated differentiations and integrations, eq (21) is reduced to the form

$$\Delta h^{\prime \prime }=B_{0}+B_{1}T^{2}+B_{2}p$$ (22)

where *p* is the saturation vapor pressure in Pa at temperature *T, P_a_* = 101325 Pa and *p_i_* = 611 Pa. The coefficients are given in table 2. Substitution of eqs (20) and (22) into (18) yields

$$\begin{array}{l} h^{\prime \prime }={h^{\prime \prime }}_{i}+A_{0}(T-T_{i})+\frac{A_{1}}{2}(T^{2}-T_{i}^{2})\\ +\frac{A_{2}}{3}(T^{3}-T_{i}^{3})+\Delta h^{\prime \prime }. \end{array}$$ (23)

A numerical value remains to be assigned to the reference enthalpy *h*″*_i_.* At any specified temperature *T*, the latent heat of fusion of ice *l*″ is given by

$$l^{\prime \prime }=h^{\prime }-h^{\prime \prime }$$ (24)

where *h*′ and *h*″ are the specific enthalpies of liquid water and ice, respectively. By adopting the convention *h′_i_* = 0 at the ice-point temperature it follows that *l″_i_ = −h"_i_.* The choice of this convention will not effect the final results because the arbitrary assignment will cancel out in the computations. Use is now made of the experimentally determined value for the latent heat of fusion of ice at 0 °C and standard atmospheric pressure recommended by Osborne [36]^5^, namely, 333.535 J/g. By means of the thermodynamic relationship

$${\left(\frac{\partial h}{\partial P}\right)}_{T}=v-T{\left(\frac{dv}{dT}\right)}_{P}$$ (25)

the latent heat was adjusted from standard atmospheric pressure (101325 Pa) to the saturation vapor pressure of ice at 0 °C, i.e., 611 Pa, yielding *l*″*_i_ = −h*″*_i_* = 333.430 J/g. Equation (23) therefore becomes

$$\begin{array}{r} h^{\prime \prime }=-{l^{\prime \prime }}_{i}+A_{0}(T-T_{i})+\frac{A_{1}}{2}\left(T^{2}-T_{i}^{2}\right)\\ +\frac{A_{2}}{3}(T^{3}-T_{i}^{3})+\Delta h^{\prime \prime } \end{array}$$ (26)

### 2.5. Enthalphy of Water Vapor

From eqs (3) and (6) it follows that

$$v=\frac{RT}{p}(1+B^{\prime }p+C^{\prime }p^{2}+\cdots )$$ (27)

and

$$\begin{array}{r} {\left(\frac{\partial v}{\partial T}\right)}_{p}=\frac{R}{p}(1+B^{\prime }p+C^{\prime }p^{2}+\cdots )\\ +\frac{RT}{p}\left(p\frac{\partial B^{\prime }}{\partial T}+p^{2}\frac{\partial C^{\prime }}{\partial T}+\cdots \right) \end{array}$$ (28)

which on substitution into eq (25) yield

$${\left(\frac{\partial h}{\partial p}\right)}_{T}=-RT^{2}\left[\frac{\partial B^{\prime }}{\partial T}+p\frac{\partial C^{\prime }}{\partial T}+\cdots \right].$$ (29)

Integration with respect to *p* leads to

$$h_{p,T}=h_{p_{0},T}-RT^{2}\frac{\partial B^{\prime }}{\partial T}p-\frac{1}{2}RT^{2}\frac{\partial C^{\prime }}{\partial T}p^{2}-\cdots$$ (30)

where *h_P,T_* is the enthalpy of water vapor at saturation pressure *p* and temperature *T* and $h_{p_{0},T}$ is the ideal-gas (zero- pressure) specific enthalpy at the same temperature *T.* The third and higher-order terms will be ignored because their contributions to *h_p,T_* are negligible. Thus, by setting

$$\Delta h=RT^{2}\frac{\partial B^{\prime }}{\partial T}p$$ (31)

the result can be written

$$h_{p,T}=h_{p_{0},T}-\Delta h.$$ (32)

Friedman and Haar [37] have calculated the ideal-gas (zero-pressure) specific heat $c_{p_{0}}/R$ for water vapor from statistical mechanical considerations over a wide range of temperatures. Their calculated values from 170 to 280 K were fitted to a polynomial equation by the method of least squares which, after multiplying by *R*, has the form

$$c_{p_{0}}=D_{0}+D_{1}T+D_{2}T^{2}+D_{3}T^{3}$$ (33)

in units of J/gK. The coefficients are given in table 2. Integrating with respect to temperature from the ice point temperature *T_i_* to *T* one gets

$$h_{p_{0},T}-h_{p_{0},T_{i}}=\int_{T_{i}}^{T}c_{p_{0}}dT$$ (34)

which becomes

$$\begin{array}{r} h_{p_{0},T}-h_{p_{0},T_{i}}=D_{0}(T-T_{i})+\frac{D_{1}}{2}(T^{2}-T_{i}^{2})\\ +\frac{D_{2}}{3}(T^{3}-T_{i}^{3})+\frac{D_{3}}{4}(T^{4}-T_{i}^{4}) \end{array}$$ (35)

where $h_{p_{0},T}$ and $h_{p_{0},T_{i}}$ are the ideal-gas (zero-pressure) specific enthalpies of water vapor at temperatures *T* and *T_i_* in units of J/g.

The ideal gas specific enthalphy $h_{p_{0},T_{i}}$ is a constant to which a numerical value must be assigned. In order to do so use is made of the latent heat of vaporization *l*′ at the ice point. By definition

$$l^{\prime }=h-h^{\prime }.$$ (36)

It will be recalled that the convention that *h′_t_* = 0 at the ice point has already been adopted. Hence at this temperature *h_i_ = l′_i_* with the result that eq (32) becomes

$$h_{i}=h_{p_{0},T_{i}}-\Delta h_{i}$$ (37)

where

$$\Delta h_{i}=RT_{i}^{2}\frac{\partial B^{\prime }}{\partial T}p_{i}.$$ (38)

Replacing *h_i_* by *l*′*_i_* in eq (37), one obtains

$$h_{p_{0},T_{i}}={l^{\prime }}_{i}+\Delta h_{i}.$$ (39)

Substituting eq (39) into eq (35) gives rise to an expression for the ideal-gas specific enthalpy of water vapor, that is,

$$\begin{array}{l} h_{p_{0},T}={l^{\prime }}_{i}+\Delta h_{i}+D_{0}(T-T_{i})+\frac{D_{1}}{2}(T^{2}-T_{i}^{2})\\ \;+\frac{D_{2}}{3}(T^{3}-T_{i}^{3})+\frac{D_{3}}{4}(T^{4}-T_{i}^{4}). \end{array}$$ (40)

Now by inserting eq (40) into (32) the real-gas specific enthalpy of saturated water vapor ensure, namely,

$$\begin{array}{l} h=h_{p,T}={l^{\prime }}_{i}+\Delta h_{i}-\Delta h+D_{0}(T-T_{i})\\ \;+\frac{D_{1}}{2}(T^{2}-T_{i}^{2})+\frac{D_{2}}{3}(T^{3}-T_{i}^{3})+\frac{D_{3}}{4}(T^{4}-T_{i}^{4}) \end{array}$$ (41)

To calculate *l*′*_i_*, use is made of an approach due to Osborne [38] which starts with the definition of an experimentally measured calorimetric quantity *γ*

$$l^{\prime }=\gamma -\delta .$$ (42)

*γ* has been quite accurately measured [38–41]. δ is given by [38]

$$\delta =\left(\frac{v^{\prime }}{v-v^{\prime }}\right)l^{\prime }=v^{\prime }T\frac{dp}{dT}$$ (43)

where *v* and *v*′ are the specific volumes of saturated vapor and water, respectively, and *dp*/*dT* is the temperature derivative of the vapor pressure of liquid water. The quantity *γ* is represented with high precision from 273.15 to 423.15 K in units of J/g by the following polynomial equation [3]

$$\gamma =F_{0}+F_{1}T+F_{2}T^{2}+F_{3}T^{3}.$$ (44)

The coefficients are given in table 2. At *T* = 273.15 K, *γ_i_* = 2500.8384 J/g. The quantity *δ_i_*, at *T* = 273.15 K, using *v*′ = 1.00016 cm^3^/g [42] and *dp*/*dT* = 44.4 Pa/K [3], is 0.0121 J/g. Therefore *l*′*_i_* = 2500.8263 J/g. By appropriate substitutions into eq (38) one obtains Δ*h_i_* = 0.2365 J/g.

### 2.6. Latent Heat of Sublimation

Substitution of eqs (26) and (41) into (2) gives rise to the following equation for the latent heat of sublimation:

$$\begin{array}{l} l=[(\gamma_{i}-\delta_{i}+\Delta h_{i}+{l^{\prime }}_{i})-(D_{0}-A_{0})T_{i}\\ \;-\frac{1}{2}(D_{1}-A_{1})T_{i}^{2}-\frac{1}{3}(D_{2}-A_{2})T_{i}^{3}-\frac{1}{4}D_{3}T_{i}^{4}]\\ \;+(D_{0}-A_{0})T+\frac{1}{2}(D_{1}-A_{1})T^{2}+\frac{1}{3}(D_{2}-A_{2})T^{3}\\ \;+\frac{1}{4}D_{3}T^{4}-\Delta h-\Delta h^{\prime \prime }. \end{array}$$ (45)

### 2.7 Vapor Pressure

Combining eqs (5) and (45), selecting the temperature *T_t_* and vapor pressure *p_t_* at the triple point as the lower limits of integration, taking any temperature *T* and corresponding vapor pressure *p* as the upper limits, and performing some simple mathematical manipulations, one obtains

$$\begin{array}{l} \int_{p_{t}}^{p}d(\ln\;p)\\ \;=\sum_{j=0}^{4}G_{j}(T^{j-1}-T_{t}^{j-1})+G_{5}\;\ln\;\left(\frac{T}{T_{t}}\right)\\ \;-\int_{T_{t}}^{T}\frac{\Delta h}{RT^{2}}dT-\int_{T_{t}}^{T}\frac{\Delta h^{\prime \prime }}{RT^{2}}dT\\ \;-\int_{T_{t}}^{T}\frac{l}{RT^{2}}\left(\frac{Z-1}{Z}\right)dT+\int_{T_{t}}^{T}\frac{l}{ZRT^{2}}\left(\frac{v^{\prime \prime }}{v}\right)dT \end{array}$$ (46)

where

$$\begin{array}{l} G_{0}=-\frac{1}{R}[\left(\gamma_{i}-\delta_{i}+\Delta h_{i}+{l^{\prime \prime }}_{i}\right)\\ \;-(D_{0}-A_{0})T_{i}-\frac{1}{2}(D_{1}-A_{1})T_{i}^{2}\\ \;-\frac{1}{3}(D_{2}-A_{2})T_{i}^{3}-\frac{1}{4}D_{3}T_{i}^{4}] \end{array}$$ (47)

$$G_{2}=\frac{D_{1}-A_{1}}{2R}$$ (48)

$$G_{3}=\frac{D_{2}-A_{2}}{6R}$$ (49)

$$G_{4}=\frac{D_{3}}{12R}$$ (50)

and

$$G_{5}=\frac{D_{0}-A_{0}}{R}$$ (51)

The coefficients are given in table 2.

The first two terms on the right-hand side of eq (46) provide the major contribution to the vapor pressure; the integrals are small corrections which account, in part, for the deviation of water vapor from ideal gas behavior. These have been left in integral form because each is a function of *p* as well as *T*.

The absolute temperature assigned to the triple point on IPTS-68 is 273.16 K. The corresponding vapor pressure is 611.657 Pa, the definitive value measured by Guildner, Johnson, and Jones [4]. The specific gas constant for water, *R* = 0.461520 J/g K, was derived from the CODATA recommended value of 8.31441 J/mol K for the universal gas constant [43], and 18.015227 g for the molar mass of naturally occurring water vapor on the unified carbon-12 scale.^6^

Because eq (46) is implicit in *p* it had to be solved by iteration. Each of the integrals on the right-hand side was evaluated at intervals of 0.25 kelvins by means of the trapezoidal rule [47]. Iteration at each interval was terminated when successive values of *p* differed by less than 0.1 ppm. The magnitudes of the terms in eq (46) are shown in skeleton form in table 3. The magnitudes of the integral terms are equivalent to the relative contributions they make to the vapor pressure. The sum of the integrals increases from zero at the triple point to −0.000389 at −100 °C. Neglecting the integrals, therefore, would introduce an error of up to 389 ppm in the vapor pressure. The sums of the integrals, at intervals of 2 kelvins, were fitted by the method of least squares to the equation

$$\sum \text{integrals}=\sum_{j=0}^{4}H_{j}\left(T^{j-1}-T_{t}^{j-1}\right)+H_{5}\;\ln\;\left(\frac{T}{T_{t}}\right)$$ (52)

with a residual standard deviation [48] of 0.7 × 10^−6^. The coefficients are given in table 2. Substituting eq (52) into (46), integrating the left-hand side, and combining terms, one finally obtains

$$\ln\;\left(\frac{p}{p_{t}}\right)=\sum_{j=0}^{4}K_{j}(T^{j-1}-T_{t}^{j-1})+K_{5}\ln\left(\frac{T}{T_{t}}\right)$$ (53)

which reduces to

$$\ln\;p=\sum_{j=0}^{4}K_{j}T^{j-1}+K_{5}\;\ln\;T$$ (54)

where

$$K_{0}=G_{0}+H_{0}$$ (55)

$$K_{1}=-\left(\frac{K_{0}}{T_{t}}+K_{2}T_{t}+K_{3}T_{t}^{2}+K_{4}T_{t}^{3}+K_{5}\ln\;T_{t}-\ln\;p_{t}\right)$$ (56)

$$K_{2}=G_{2}+H_{2}$$ (57)

$$K_{3}=G_{3}+H_{3}$$ (58)

$$K_{4}=G_{4}+H_{4}$$ (59)

$$K_{5}=G_{5}+H_{5}.$$ (60)

The coefficients are given in table 2.

The feasibility of reducing the number of terms in eqs (53) and (54) was investigated. Values of vapor pressure were generated from eq (53) at one-kelvin intervals from the ice point to 172.15 K and fitted by the method of least squares to equations of the form

$$\ln\;\left(\frac{p}{p_{t}}\right)=\sum_{j=0}^{n}L_{j}(T^{j-1}-T_{t}^{j-1})+L_{n+1}\ln\;\left(\frac{T}{T_{t}}\right)$$ (61)

for $0\bar{<}n\bar{<}4$ with and without the $\ln\;\left(\frac{T}{T_{t}}\right)$ term. For *n* = 2, with the $\ln\;\left(\frac{T}{T_{t}}\right)$ term included, the standard deviation of the fit was 14 ppm and the maximum deviation was 26 ppm. Thus, for *n* = 2, eq (61) becomes

$$\ln\;\left(\frac{p}{p_{t}}\right)=\sum_{j=0}^{2}L_{j}(T^{j-1}-T_{t}^{j-1})+L_{3}\ln\;\left(\frac{T}{T_{t}}\right)$$ (62)

or, alternately,

$$\ln\;p=\sum_{j=0}^{2}L_{j}T^{j-1}+L_{3}\;\ln\;T$$ (63)

where

$$L_{1}=-\frac{L_{0}}{T_{t}}-L_{2}T_{t}-L_{3}\;\ln\;T_{t}+\ln\;p_{t}$$ (64)

The coefficients are given in table 2.

## 3. Error Analysis

It is of interest to assign reasonable bounds of uncertainty to the independent variables and constants and then calculate the effect of these uncertainties on *p.* Start with eq (5) and recall that

$$Z=1+B^{\prime }p$$ (65)

$$v=\frac{ZRT}{p}$$ (66)

$$v^{\prime \prime }={v^{\prime \prime }}_{p_{a},T_{i}}\left[1+3\int_{T_{i}}^{T}\alpha_{P_{a}}\;dT\right]\left[1-k(p-P_{a})\right]$$ (67)

$$l=\int_{T_{i}}^{T}c_{p_{o}}dT+\gamma_{i}-\delta_{i}+\Delta h_{i}-\Delta h-\int_{T_{i}}^{T}{c^{\prime \prime }}_{P_{a}}\;dT-\Delta h^{\prime \prime }+{l^{\prime \prime }}_{i}$$ (68)

where

$$\Delta h=RT^{2}\frac{\partial B^{\prime }}{\partial T}p$$ (69)

and

$$\begin{array}{l} \Delta h^{\prime \prime }=\int_{p_{i}}^{P_{a}}{\left[v^{\prime \prime }-T{\left(\frac{\partial v^{\prime \prime }}{\partial T}\right)}_{p_{i}}\right]}_{T_{i}}dP\\ \;+\int_{P_{a}}^{p}{\left[v^{\prime \prime }-T{\left(\frac{\partial v^{\prime \prime }}{\partial T}\right)}_{P_{a}}\right]}_{T}dP \end{array}$$ (70)

Substituting the above equations into eq (5) converts the latter into a functional relationship of independent variables and constants. The vapor pressure is calculated by iteration and numerical integration, as previously described. The calculation then is repeated with each variable and constant separately augmented by its appropriate estimated error.

The absolute temperature *T* enters into eq (5) as the independent variable so that it is subject neither to experimental nor scale error. However, experimental and scale errors in the temperature affect the uncertainties in the independent variables; therefore, these temperature errors are contained in the estimated errors of the independent variables. Since *T_t_* is assigned values on IPTS-68, it will be assumed that its estimated uncertainty is zero.

The estimated error in the specific gas constant for water vapor *R* arises from the assigned (three standard deviations) uncertainty [43] in the molar gas constant of 78 × 10^−5^ J/mol K and from a calculated (three standard deviations) uncertainty in the molecular weight of naturally occurring water of 9 × 10^−5^ g/mol based on the assigned uncertainties [46] in the relative atomic masses of the pertinent nuclides. The resultant estimated error (three standard deviations) in *R* is 45 × 10^−6^ J/g K (94 ppm).

There are no experimental data below 273.15 K on which to base an estimate of the uncertainty in the virial coefficient B′ nor in the derivative *dB*′/*dT.* Therefore, four sets of extrapolated virial coefficients were calculated, using the empirical equations of Goff and Gratch [12–14], Keyes [15, 16], and Juza as given by Bain [17], and then differences were obtained from the latest Keyes values [16]. The estimated uncertainty was set at thrice the maximum difference. This uncertainty in *B*′ contributed to a corresponding uncertainty in *dB*′/*dT.*

*P_a_* is standard atmospheric pressure. Because this is an assigned value it will be assumed that its uncertainty is zero.

Guildner, Johnson, and Jones [4] have assigned an estimated uncertainty (three standard deviations plus systematic errors) of 0.010 Pa (16 ppm) to their measured value of the vapor pressure at the triple point *p_t_.* Their estimated uncertainty will be used here.

According to Ginnings and Corruccini [19], the combined random and systematic uncertainty in their determination of the density of ice at 0 °C and 1 atmosphere is 0.00005 g/ml. This value was converted to 0.00006 cm^3^/g and the latter used as the estimated uncertainty in the specific volume of ice ${v^{\prime \prime }}_{p_{a},T_{i}}$. The estimated uncertainty in the coefficient $\alpha_{P_{a}}$ will be taken as three times the standard deviation of the fit [48] as given by eq (10), that is, 0.50 × 10^−5^ cm/cm K. Leadbetter [27] has ascribed an uncertainty of 5 percent to his values of the adiabatic compressibility of ice, namely 0.6 cm^3^/cm^3^ Pa. The same uncertainty is therefore used for the isothermal compressibility *k*, since the latter is derived from Leadbetter’s values.

Friedman and Haar [37] have computed $c_{p_{0}}/R$ to six significant figures. However, they did not give an estimate of the uncertainty in their calculated values. An error of 100 ppm therefore was assigned to $c_{p_{0}}/R$. Combining this error along with 99 ppm for the estimated uncertainty in *R* and 9 ppm which represents three times the residual standard deviation of the fit of eq (33) resulted in an estimated error of 140 ppm in $c_{p_{0}}$, i.e., 0.26 × 10^−3^ J/g K.

In the absence of any other criteria for estimating the uncertainty in ${c^{\prime \prime }}_{P_{a}}$, a value of 0.0103 J/g K was selected which equals three times the standard deviation of the fit of eq (19), 0.0099 J/g K, plus an estimated error of 0.0004 J/g K due to ambiguities in the temperature scale employed by Giauque and Stout.

The estimated error in *γ_i_* was taken as 0.45 J/g which is three times the standard deviation of the fit of eq (44). The uncertainty in *δ_i_* was conservatively estimated at less than one percent, that is, less than 0.0001 J/g. Osborne [36] has estimated that the random and systematic error in $l_{i}^{\prime \prime }$ was 0.2 J/g and his value, therefore, was used here.

The quantity Δ*h*″ varies from zero at 0 °C to about −0.002 J/g at −100 °C. Since it is small compared to *l* (~ 2830 J/g), its functional dependence on other parameters will be ignored. The uncertainty in Δ*h*″ was estimated at less than 0.0001 J/g.

A summary of the individual estimated errors contributing to the total error in the predicted vapor pressure is given in table 4. The corresponding uncertainty in *p* due to each of the enumerated errors is shown in table 5. The square root of the sum of the squares of the individual errors was used as the best estimate of the overall maximum error in *p* [49]. As the temperature decreases from the triple point to −100 °C, the estimated relative error in *p* increases from 16 ppm to 0.5 percent.

## 4. Comparisons

The first experimental values of the vapor pressure of ice were reported by Regnault [50] in 1847. Subsequently, measurements were made by Fischer [51], Juhlin [52], and Marvin [53]. In 1909, Scheel and Heuse [54] at the Physikalisch-Technische Reichsanstalt (PTR) published the results of their work which superseded all earlier determinations for range, precision and accuracy. Using a Rayleigh inclined manometer and a platinum resistance thermometer they measured the vapor pressure from 0 to −67 °C. In a second paper [55] they suggested that temperatures interpolated from the Callendar formula would be more in accord with the thermodynamic scale than the temperatures given in their first paper. In 1919, the PTR issued revised values of the Scheel and Heuse measurements [56]. Although not explicitly stated, these new values appear to have been based on the use of the Callendar formula for interpolating temperature measurements with platinum resistance thermometers.

Weber [57] in 1915, employing both a hot-wire manometer and a Knudsen radiometer, made measurements from −22 to −98 °C. A limited number of determinations were made by Nernst [58] in 1909 and by Drucker, Jimeno, and Kangro [59] in 1915. Douslin and McCullough [60] in 1963, using an inclined dead-weight piston gage, made measurements to −30 °C. Jancso, Pupezin, and Van Hook [61] in 1970 used a differential capacitance manometer to effect a series of determinations to −78 °C. They used the vapor pressure of ice at 0 °C as the reference pressure for their manometer, assigning to it the value 4.581 mm Hg (610.7 Pa).

**Figure 1:**
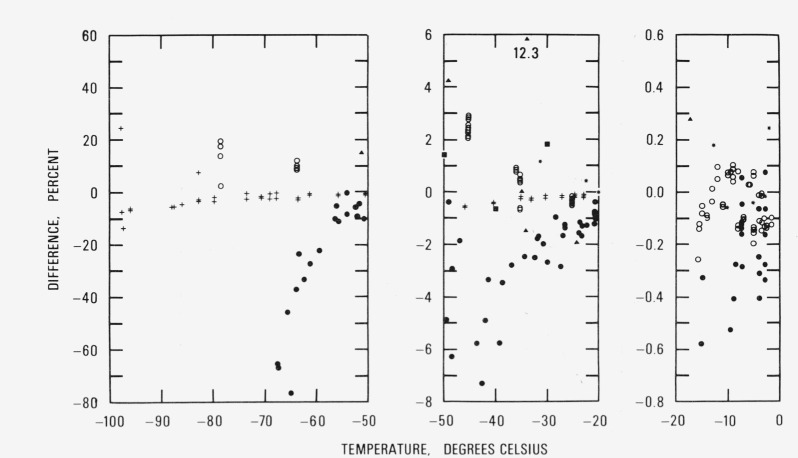
Comparision with vapor pressure measurements. Relative vapor pressure difference $\left[\frac{\text{measurement}-\text{eq}(54)}{\text{eq}(54)}\times 100\right]$ between measurement and eq (54) in percent: ● Scheel and Heuse; + Weber; ■ Nernst; ▲ Drucker, Jimeno and Kangro; * Douslin and McCullough; ○ Jancso, Pupezin and Van Hook.

A comparison of eq (54) with these measurements, excluding the early work of Regnault, Fischer, Juhlin, and Marvin, is shown in figure 1. The temperatures given by the investigators were converted to IPTS-68 for this comparison. Many of the errors associated with these measurements are not given explicitly so it is difficult to determine both their sources and magnitudes. Therefore, no attempt has been made to assign uncertainties nor to make corrections except for the temperature scale and, where noted, for reference pressure. Because the Jancso, Pupezin and Van Hook pressure measurements were made with respect to the vapor pressure at the ice point they were adjusted to conform to the vapor pressure at 0 °C predicted by eq (54), namely, 611.154 Pa rather than 610.7 Pa (4.581 mm Hg) which Jancso, Pupezin, and Van Hook used.

The sets of data of some of the investigators tend to deviate from eq (54) in consistent ways. The Scheel and Heuse measurements (black dots) are generally lower in magnitude (except for two points) than the vapor pressures calculated from eq (54); the differences increase until at −67 °C they are of the order of 70 percent. Weber’s measurements (pluses) are much closer, but they also are lower in magnitude (except for two points); at about −98 °C, where suprisingly Weber obtained several measurements, the deviations are as large as 25 percent. Among all the investigators, the best agreement is achieved with Weber. However, Weber made no measurements above −22 °C.

Of the three measurements of Nernst (black squares) two (at −30 and −50 °C) show positive differences and the third (at −40 °C) a negative difference, none exceeding 2 percent. The Drucker, Jimeno, and Kangro measurements (black triangles) tend to be high, with one value (at −34 °C) differing by as much as +12.3 percent. The differences for the Douslin and McCullough measurements (asterisks), which cover the range of temperature from −2 to −31.4 °C, are almost equally positive and negative in number and reach a magnitude of about one percent at −31.4 °C. The Jancso, Pupezin, and Van Hook differences (circles) scatter more or less randomly in the temperature region above −15 °C; from −35 °C and below, the differences are all positive, reaching a magnitude of 20 percent at about −78 °C.

The differences far exceed the estimated uncertainty of the values predicted by eq (54). It may be inferred from the difference patterns displayed by these several sets of data that there are significant systematic errors present in each of these data. The obvious conclusion is that a definitive set of measurements remains to be made.

Numerous empirical equations have been proposed to represent the vapor pressure of ice. Scheel and Heuse [54] and Thiesen [62] derived formulas which fit the original Scheel and Heuse data [54]. The equations of Tetens [63] and Erdelyszky as given by Sonntag [64], are of the Magnus type [65] with different sets of coefficients. The Jancso, Pupezin, and Van Hook [61] empirical equation is based on a least square fit to their own measurements.

There also have been repeated attempts to derive thermodynamically based expressions for the vapor pressure of ice. The equations of Nernst [58], Washburn [66], Whipple [67], and Goff and Gratch [68, 69] were obtained by integrating the Clausius-Clapeyron equation and inserting selected values of thermal data. Vapor pressures based on the Nernst equation were included in an early edition of the Smithsonian Meteorological Tables [70]. Vapor pressures based on the Washburn equation are given in several standard references [71, 72] often used by chemists. The Goff formulation is used in the meteorological and air conditioning disciplines [73–75]. The equation ascribed to Kelley [76] is based on an expression he derived for the free energy difference which, when integrated with respect to temperature, yields the logarithm of the vapor pressure. This equation is given in a widely used set of German tables [77] and by Dushman [78]. The equations of Miller [79] and Jancso, Pupezin, and Van Hook [61] were derived from an expression for the vaporization process given in terms of vapor fugacity and condensed phase activity [80]. The Miller equation was not presented in explicit form although calculated vapor pressures were given in an abbreviated table.

**Figure 2:**
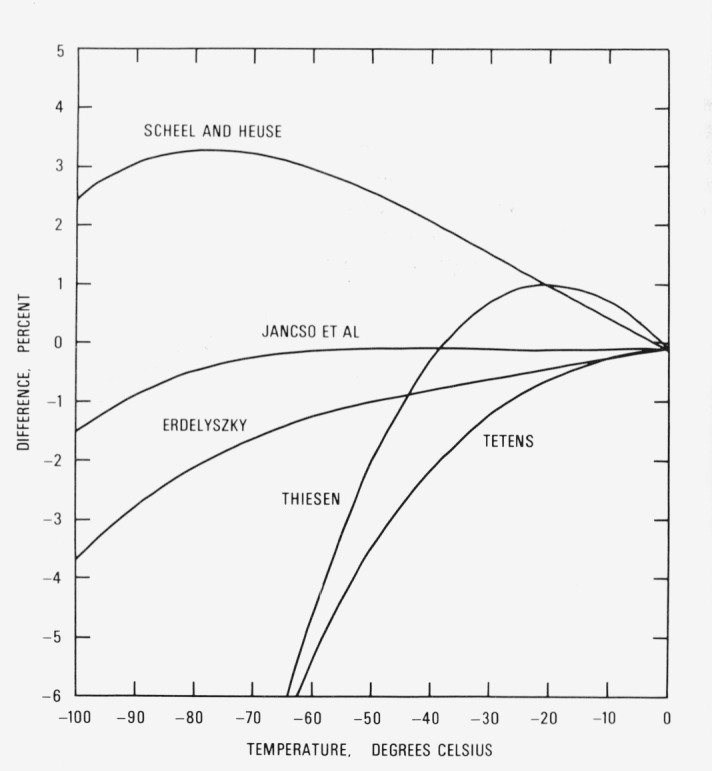
Comparison with empirical equations. Relative vapor pressure difference $\left[\frac{\text{other}-\text{eq}(54)}{\text{eq}(54)}\times 100\right]$ between empirical equation cited in the literature and eq (54) in percent.

**Figure 3:**
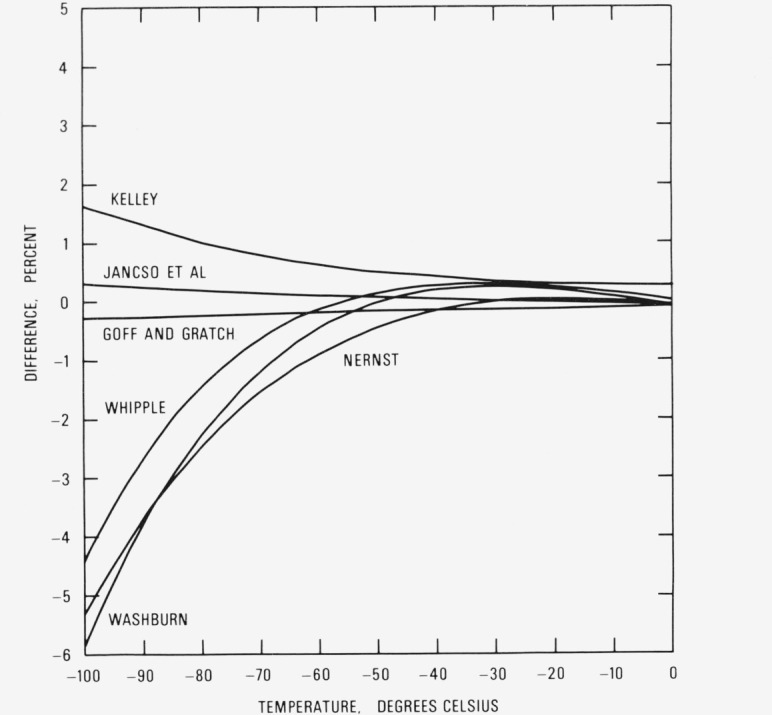
Comparison with thermodynamic equations. Relative vapor pressure difference $\left[\frac{\text{other}-\text{eq}(54)}{\text{eq}(54)}\times 100\right]$ between thermodynamic equation cited in the literature and eq (54) in percent.

A comparison between the empirical equations and eq (54) is shown in figure 2 and a similar comparison between the thermodynamic equations and eq (54) is shown in figure 3. Because the Thiesen and the Whipple equations give functional relationships for the ratio *p*/*p*_0_, where *p* is the vapor pressure at any given temperature and *p*_0_ is the vapor pressure at 0 °C, the value predicted by eq (54) was inserted for *p*_0_ to compute *p* rather than the value used by these investigators. No attempt was made to adjust or correct any of the empirical equations from the temperature scale used by the investigator in his formulation to IPTS-68.

All the empirical formulations, except that of Jancso, Pupezin, and Van Hook, deviate substantially from eq (54). This, in part, may be accounted for by errors in the temperature scale. More important, however, is the fact that these equations were fitted to experimental data and it has already been demonstrated (see fig. 1) that there are significant differences between those data and eq (54). On the other hand, the Jancso, Pupezin, and Van Hook data differ randomly from eq (54) above −15 °C. Therefore, it is reasonable to expect their empirical equation to agree closely with eq (54) in this region, as indeed it does. What is not clear is why at lower temperatures, say from −50 to −80 °C, the differences between their equation and eq (54) are negative whereas the differences between their measurements and eq (54) are positive. No significance is attached to the differences below −80 °C because their equation was not fitted to data at these lower temperatures and hence is an extrapolation.

There is much better accord between the thermodynamic equations and eq (54), at least down to about −40 °C. Below −40 °C the Kelley, Whipple, Nernst, and Washburn equations deviate increasingly from eq (54); at −100 °C, they differ from eq (54) by +1.6, −4.4, −5.4, and −5.8 percent, respectively. There is good agreement between the Goff and Gratch equation and eq (54); the former yields calculated values that are smaller by 0.08 percent at 0 °C and by 0.29 percent at −100 °C. There is also good agreement between the Jancso, Pupezin, and Van Hook equation and eq (54); the vapor pressures from their thermodynamic calculations are smaller by 0.06 percent at 0 °C but are larger by 0.31 percent at −100 °C. The vapor pressures from the Goff and Gratch equation and the Jancso, Pupezin and Van Hook equation straddle both sides of those derived from eq (54).

## 5. Tabulations

Vapor pressures were computed from eq (54) and are given in pascals as a function of temperature (in degrees Celsius on the IPTS-68 scale) at 0.1-degree intervals from 0 to −100 °C. These computed values, as well as the derivative with respect to temperature at intervals of 1 degree C, are given in table 6.

## 6. Discussion

Two equations are offered for use by those who wish to compute the vapor pressure rather than to select or interpolate it from tabulated values. Equation (54) is the preferred equation because it has a rational thermodynamic basis. If a simpler form is desired, then eq (63) may be used, but it should be remembered that the latter equation is empirical. Although the vapor pressures in table 6 are given to six significant figures, the accuracy ascribed to these values is no better than that listed in table 5. Finally, because of the truncating procedure used in the calculation, the last significant figure may differ by 1 from the best rounded value.

## Figures and Tables

**Table 1:** Compressibility factor for saturated water vapor over ice

Temperature	Compressibility Factor*a*			

°C	Keyes 1969*b* Z	Keves 1947*c* Z	Bain*d* Z	Goff and Gratch*e* Z
0.01	0.999624	0.999501	0.999529	0.999506
0	.999624	.999501	.999529	.999506
−10	.999907	.999726	.999747	.999730
−20	.999958	.999856	.999871	.999859
−30	.999982	.999928	.999938	.999930
−40	.999993	.999966	.999972	.999967
−50	.999998	.999984	.999986	.999985
−60	.999999	.999994	.999996	.999994
−70	1.000000	.999997	.999999	.999998
−80	1.000000	.999999	1.000000	.999999
−90	1.000000	1.000000	1.000000	1.000000
−100	1.000000	1.000000	1.000000	1.000000

*^a^*Calculated by eq (6) using *B*′ given by the indicated investigator.

*^b^*Ref [16].

*^c^*Ref [15].

*^d^*Ref [17].

*^e^*Ref [12–14].

**Table 2:** Coefficients to equations

*j*						

	0	1	2	3	4	5
						
A	0.274292	0.582109 × 10^−2^	0.325031 × 10^−5^			
B	−0.344 × 10^−2^	.3765 × 10^−7^	.107 × 10^−5^			
D	.1834928 × 10^1^	.2542981 × 10^−3^	−0.15852458 × 10^−5^	0.3550699 × 10^−8^		
F	.34238440 × 10^4^	−0.52277204 × 10^1^	.98557190 × 10^−2^	−0.11305118 × 10^−4^		
G	−0.57284294 × 10^4^		−0.60309325 × 10^−2^	−0.17462428 × 10^−5^	0.64112408 × 10^−9^	0.33815137 × 10^1^
H	−0.1369402 × 10^3^		.19779974 × 10^−1^	−0.32285532 × 10^−4^	.26326563 × 10^−7^	−0.26896486 × 10^1^
K	−0.58653696 × 10^4^	.2224103300 × 10^2^	.13749042 × 10^−1^	−0.34031775 × 10^−4^	.26967687 × 10^−7^	.6918651
L	−0.57170491 × 10^4^	.9158658955 × 10^1^	−0.74950412 × 10^−2^	0.36067657 × 10^1^		

**Table 3:** Magnitude of the terms in eq (46)

*t*	$\sum_{j=0}^{4}G_{j}\left(T^{j-1}-T_{t}^{j-1}\right)+G_{5}\ln\;\left(\frac{T}{T_{t}}\right)$	$\int_{T_{t}}^{T}\frac{\Delta h}{RT^{2}}dT$	$\int_{T_{t}}^{T}\frac{\Delta h\prime \prime }{RT^{2}}dT$	$\int_{T_{t}}^{T}\frac{l}{RT^{2}}\left(\frac{Z-1}{Z}\right)dT$	$\int_{T_{t}}^{T}\frac{l}{ZRT^{2}}\left(\frac{v\prime \prime }{v}\right)dT$	$\sum_{\text{integrals}}$	$\ln\;\left(\frac{p}{p_{t}}\right)$	*P*
								
°C								Pa
								
0.01	−0.0000000	−0.0000000	0.0000000	0.0000000	−0.0000000	−0.0000000	−0.0000000	611.657
0	−0.0008231	−0.0000000	.0000000	.0000001	−0.0000000	−0.0000001	−0.0008232	611.154
−10	−0.8556016	−0.0000515	.0000001	.0002348	−0.0000031	−0.0001864	−0.8557880	259.923
−20	−1.7784775	−0.0000786	.0000003	.0003614	−0.0000045	−0.0002877	−1.7787652	103.276
−30	−2.7776170	−0.0000920	.0000007	.0004258	−0.0000051	−0.0003397	−2.7779567	38.0239
−40	−3.8625689	−0.0000982	.0000012	.0004565	−0.0000054	−0.0003649	−3.8629338	12.8486
−50	−5.0445714	−0.0001008	.0000018	.0004701	−0.0000055	−0.0003766	−5.0449480	3.94017
−60	−6.3369461	−0.0001018	.0000026	.0004756	−0.0000055	−0.0003818	−6.3373279	1.08204
−70	−7.7556071	−0.0001022	.0000035	.0004776	−0.0000055	−0.0003844	−7.7559915	0.261893
−80	−9.3197265	−0.0001023	.0000045	.0004783	−0.0000055	−0.0003860	−9.3201125	.0548068
−90	−11.0526186	−0.0001024	.0000058	.0004784	−0.0000055	−0.0003874	−11.0530060	.00968832
−100	−12.9829316	−0.0001024	.0000074	.0004785	−0.0000055	−0.0003890	−12.9833206	.00140580

$\sum_{\text{integrals}}=-\int_{T_{t}}^{T}\frac{\Delta h}{RT^{2}}dT-\int_{T_{t}}^{T}\frac{\Delta h\prime \prime }{RT^{2}}dT-\int_{T_{t}}^{T}\frac{l}{RT^{2}}\left(\frac{Z-1}{Z}\right)dT+\int_{T_{t}}^{T}\frac{l}{ZRT^{2}}\left(\frac{v\prime \prime }{v}\right)dT$

**Table 4:** Summary of estimated errors in variables and constants

Temperature	Parameter											
*t*	*R*		*B*′		*P_t_*		${v^{\prime \prime }}_{P_{a},T_{i}}$		*k*		$c_{p_{o}}$	
°C	Magnitude J/gK	Error J/gK	Magnitude 1/Pa	Error 1/Pa	Magnitude Pa	Error Pa	Magnitude em^3^/g	Error cm^3^/g	Magnitude cm^3^/cm^3^ Pa	Error cm^3^/cm^3^Pa	Magnitude J/gK	Error J/gK
												
0.01	0.461520	0.000045	−0.6151 × 10^−6^	0.6303 × 10^−6^	611.657	0.010	1.09089	0.00006	0.13 × 10^−9^	0.6 × 10^−11^	1.85848	0.00026
0	.461520	.000045	−0.6151 × 10^−6^	.6303 × 10^−6^	611.657	.010	1.09089	.00006	.13 × 10^−9^	.6 × 10^−11^	1.85848	.00026
−10	.461520	.000045	−0.7420 × 10^−6^	.9333 × 10^−6^	611.657	.010	1.09089	.00006	.13 × 10^−9^	.6 × 10^−11^	1.85677	.00026
−20	.461520	.000045	−0.9036 × 10^−6^	.1432 × 10^−5^	611.657	.010	1.09089	.00006	.13 × 10^−9^	.6 × 10^−11^	1.85532	.00026
−30	.461520	.000045	−0.1112 × 10^−5^	.2278 × 10^−5^	611.657	.010	1.09089	.00006	.13 × 10^−9^	.6 × 10^−11^	1.85408	.00026
−40	.461520	.000045	−0.1383 × 10^−5^	.3755 × 10^−5^	611.657	.010	1.09089	.00006	.13 × 10^−9^	.6 × 10^−11^	1.85305	.00026
−50	.461520	.000045	−0.1741 × 10^−5^	.6414 × 10^−5^	611.657	.010	1.09089	.00006	.13 × 10^−9^	.6 × 10^−11^	1.85219	.00026
−60	.461520	.000045	−0.2219 × 10^−5^	.1135 × 10^−4^	611.657	.010	1.09089	.00006	.13 × 10^−9^	.6 × 10^−11^	1.85149	.00026
−70	.461520	.000045	−0.2866 × 10^−5^	.2083 × 10^−4^	611.657	.010	1.09089	.00006	.13 × 10^−9^	.6 × 10^−11^	1.85093	.00026
−80	.461520	.000045	−0.3753 × 10^−5^	.3961 × 10^−4^	611.657	.010	1.09089	.00006	.13 × 10^−9^	.6 × 10^−11^	1.85049	.00026
−90	.461520	.000045	−0.4987 × 10^−5^	.7806 × 10^−4^	611.657	.010	1.09089	.00006	.13 × 10^−9^	.6 × 10^−11^	1.85014	.00026
−100	.461520	.000045	−0.6728 × 10^−5^	.1594 × 10^−3^	611.657	.010	1.09089	.00006	.13 × 10^−9^	.6 × 10^−11^	1.84987	.00026

Temperature	Parameter											
*t*	${c^{\prime \prime }}_{P_{a}}$		*γ_i_*		*δ_i_*		*l″_i_*		Δ*h*″		$\alpha_{P_{a}}$	
°C	Magnitude J/gK	Error J/gK	Magnitude J/g	Error J/g	Magnitude J/g	Error J/g	Magnitude J/g	Error J/g	Magnitude J/g	Error J/g	Magnitude cm/cm K	Error cm/cm K
												
0.01	2.1069	0.0103	2800.84	0.45	0.0121	0.0001	333.43	0.2	−0.0000	0.0001	0.544 × 10^−4^	0.50 × 10^−5^
0	2.1068	0.0103	2800.84	0.45	0.0121	0.0001	333.43	0.2	−0.0000	0.0001	0.544 × 10^−4^	0.50 × 10^−5^
−10	2.0312	0.0103	2800.84	0.45	0.0121	0.0001	333.43	0.2	−0.0006	0.0001	0.521 × 10^−4^	0.50 × 10^−5^
−20	1.9562	0.0103	2800.84	0.45	0.0121	0.0001	333.43	0.2	−0.0009	0.0001	0.499 × 10^−4^	0.50 × 10^−5^
−30	1.8818	0.0103	2800.84	0.45	0.0121	0.0001	333.43	0.2	−0.0012	0.0001	0.476 × 10^−4^	0.50 × 10^−5^
−40	1.8082	0.0103	2800.84	0.45	0.0121	0.0001	333.43	0.2	−0.0014	0.0001	0.453 × 10^−4^	0.50 × 10^−5^
−50	1.7351	0.0103	2800.84	0.45	0.0121	0.0001	333.43	0.2	−0.0016	0.0001	0.430 × 10^−4^	0.50 × 10^−5^
−60	1.6627	0.0103	2800.84	0.45	0.0121	0.0001	333.43	0.2	−0.0017	0.0001	0.408 × 10^−4^	0.50 × 10^−5^
−70	1.5910	0.0103	2800.84	0.45	0.0121	0.0001	333.43	0.2	−0.0019	0.0001	0.385 × 10^−4^	0.50 × 10^−5^
−80	1.5199	0.0103	2800.84	0.45	0.0121	0.0001	333.43	0.2	−0.0020	0.0001	0.362 × 10^−4^	0.50 × 10^−5^
−90	1.4494	0.0103	2800.84	0.45	0.0121	0.0001	333.43	0.2	−0.0022	0.0001	0.340 × 10^−4^	0.50 × 10^−5^
−100	1.3797	0.0103	2800.84	0.45	0.0121	0.0001	333.43	0.2	−0.0023	0.0001	0.317 × 10^−4^	0.50 × 10^−5^

**Table 5:** Summary of equivalent errors in vapor pressure due to estimated errors in variables and constants

Temperature	Parameter												Estimated Overall Error*a*
*t*	*R*	*B*′	*P_t_*	${v^{\prime \prime }}_{P_{a},T_{i}}$	*k*	$c_{p_{o}}$	${c^{\prime \prime }}_{P_{a}}$	*γ_i_*	*δ_i_*	*l″_i_*	Δ*h*″	$\alpha_{P_{a}}$	
°C	Estimated error in vapor pressure due to estimated error in indicated parameter, ppm												ppm
0.01	0	0	16	<1	<1	0	0	0	<1	0	<1	<1	16
0	0	0	16	<1	<1	0	0	0	<1	0	<1	<1	16
−10	83	532	16	<1	<1	0	15	135	<1	60	<1	<1	559
−20	173	950	16	<1	<1	1	66	282	<1	125	<1	<1	1016
−30	270	1289	16	<1	<1	3	157	440	<1	195	<1	<1	1411
−40	376	1580	16	<1	<1	7	295	612	<1	272	<1	<1	1781
−50	491	1845	16	<1	<1	12	488	799	<1	355	<1	<1	2156
−60	618	2101	16	<1	<1	18	746	1004	<1	436	<1	<1	2561
−70	756	2363	16	<1	<1	27	1081	1229	<1	546	<1	<1	3022
−80	909	2641	16	<1	<1	38	1508	1477	<1	656	<1	<1	3562
−90	1078	2943	16	<1	<1	52	2043	1752	<1	778	<1	<1	4064
−100	1278	3279	16	<1	<1	72	2708	2055	<1	912	<1	<1	4978

*^a^*Square root of the sum of the squares of the estimated errors contributed by each parameter.

**Table 6:** Saturation vapor pressure over ice

Deg	0.0	0.1	0.2	0.3	0.4	0.5	0.6	0.7	0.8	0.9	Derivative

C	Pa	Pa	Pa	Pa	Pa	Pa	Pa	Pa	Pa	Pa	Pa/K

−0	611.153	606.140	601.164	596.225	591.323	586.458	581.630	576.837	572.081	567.360	48.7738
−1	562.675	558.025	553.411	548.830	544.285	539.774	535.297	530.853	526.444	522.067	45.2441
−2	517.724	513.414	509.136	504.891	500.679	496.498	492.349	488.232	484.146	480.091	41.9451
−3	476.068	472.075	468.112	464.180	460.278	456.406	452.564	448.751	444.968	441.213	38.8634
−4	437.488	433.791	430.123	426.483	422.871	419.287	415.731	412.202	408.700	405.226	35.9864
−5	401.779	398.358	394.964	391.597	388.256	384.940	381.651	378.387	375.149	371.936	33.3021
−6	368.748	365.585	362.446	359.333	356.244	353.179	350.138	347.121	344.128	341.158	30.7990
−7	338.212	335.289	332.389	329.512	326.658	323.826	321.017	318.230	315.465	312.722	28.4662
−8	310.001	307.302	304.624	301.967	299.332	296.717	294.124	291.551	288.998	286.467	26.2936
−9	283.955	281.464	278.992	276.540	274.108	271.696	269.303	266.929	264.575	262.239	24.2713
−10	259.922	257.624	255.345	253.084	250.841	248.617	246.410	244.222	242.051	239.898	22.3900
−11	237.762	235.644	233.543	231.459	229.393	227.343	225.310	223.293	221.293	219.309	20.6412
−12	217.342	215.391	213.456	211.537	209.633	207.745	205.873	204.017	202.175	200.349	19.0163
−13	198.538	196.742	194.961	193.194	191.442	189.705	187.982	186.274	184.579	182.899	17.5077
−14	181.233	179.581	177.942	176.318	174.706	173.109	171.524	169.953	168.396	166.851	16.1079
−15	165.319	163.800	162.294	160.801	159.320	157.852	156.396	154.952	153.521	152.101	14.8099
−16	150.694	149.299	147.915	146.544	145.184	143.835	142.498	141.173	139.858	138.555	13.6070
−17	137.263	135.982	134.713	133.453	132.205	130.968	129.741	128.524	127.318	126.123	12.4932
−18	124.938	123.763	122.598	121.443	120.298	119.163	118.038	116.923	115.817	114.721	11.4624
−19	113.634	112.557	111.489	110.431	109.381	108.341	107.310	106.288	105.275	104.271	10.5091
−20	103.276	102.289	101.311	100.341	99.3809	98.4284	97.4843	96.5485	95.6210	94.7016	9.62823
−21	93.7904	92.8872	91.9920	91.1047	90.2253	89.3537	88.4898	87.6336	86.7850	85.9439	8.81467
−22	85.1104	84.2842	83.4655	82.6540	81.8498	81.0528	80.2629	79.4801	78.7043	77.9355	8.06388
−23	77.1735	76.4184	75.6701	74.9286	74.1937	73.4655	72.7438	72.0286	71.3199	70.6176	7.37151
−24	09.9217	69.2321	68.5487	67.8716	67.2005	66.5356	65.8768	65.2239	64.5770	63.9360	6.73347
−25	63.3008	62.6715	62.0479	61.4300	60.8178	60.2112	59.6101	59.0146	58.4245	57.8399	6.14595
−26	57.2607	56.6868	56.1182	55.5548	54.9966	54.4436	53.8958	53.3530	52.8152	52.2824	5.60533
−27	51.7546	51.2317	50.7136	50.2003	49.6919	49.1882	48.6892	48.1948	47.7051	47.2199	5.10825
−28	46.7393	46.2632	45.7916	45.3244	44.8616	44.4031	43.9489	43.4991	43.0534	42.6120	4.65155
−29	42.1748	41.7417	41.3126	40.8877	40.4667	40.0498	39.6368	39.2278	38.8226	38.4213	4.23227
−30	38.0238	37.6301	37.2402	36.8540	36.4714	36.0926	35.7173	35.3457	34.9776	34.6131	3.84764
−31	34.2521	33.8945	33.5404	33.1897	32.8423	32.4983	32.1577	31.8203	31.4862	31.1554	3.49509
−32	30.8277	30.5032	30.1819	29.8637	29.5486	29.2365	28.9275	28.6215	28.3185	28.0185	3.17218
−33	27.7214	27.4272	27.1358	26.8474	26.5617	26.2789	25.9988	25.7215	25.4469	25.1751	2.87668
−34	24.9059	24.6394	24.3755	24.1142	23.8555	23.5993	23.3457	23.0947	22.8461	22.5999	2.60647
−35	22.3563	22.1150	21.8762	21.6397	21.4056	21.1739	20.9444	20.7173	20.4924	20.2698	2.35960
−36	20.0494	19.8312	19.6152	19.4014	19.1898	18.9803	18.7729	18.5675	18.3643	18.1631	2.13424
−37	17.9640	17.7669	17.5717	17.3786	17.1874	16.9982	16.8108	16.6254	16.4419	16.2603	1.92868
−38	16.0805	15.9025	15.7264	15.5521	15.3795	15.2088	15.0397	14.8725	14.7069	14.5430	1.74136
−39	14.3809	14.2204	14.0615	13.9043	13.7488	13.5948	13.4424	13.2916	13.1424	12.9947	1.57080
−40	12.8486	12.7040	12.5609	12.4192	12.2791	12.1404	12.0032	11.8674	11.7330	11.6000	1.41564
−41	11.4685	11.3383	11.2095	11.0820	10.9559	10.8311	10.7076	10.5854	10.4645	10.3449	1.27461
−42	10.2266	10.1095	9.99366	9.87903	9.76563	9.65343	9.54243	9.43260	9.32395	9.21646	1.14655
−43	9.11011	9.00490	8.90082	8.79785	8.69598	8.59521	8.49552	8.39690	8.29934	8.20283	1.03036
−44	8.10736	8.01292	7.91950	7.82708	7.73567	7.64525	7.55580	7.46733	7.37981	7.29325	0.925056
−45	7.20763	7.12294	7.03917	6.95631	6.87436	6.79330	6.71313	6.63384	6.55542	6.47785	.829693
−46	6.40114	6.32526	6.25022	6.17601	6.10262	6.03003	5.95824	5.88725	5.81704	5.74761	.743420
−47	5.67894	5.61104	5.54389	5.47749	5.41182	5.34688	5.28267	5.21917	5.15638	5.09429	.665446
−48	5.03290	4.97219	4.91216	4.85280	4.79411	4.73608	4.67870	4.62196	4.56587	4.51040	.595041
−49	4.45556	4.40134	4.34773	4.29473	4.24233	4.19052	4.13930	4.08866	4.03860	3.98910	.531534
−50	3.94017	3.89179	3.84397	3.79669	3.74996	3.70375	3.65808	3.61293	3.56829	3.52417	.474306
−51	3.48056	3.43744	3.39483	3.35270	3.31106	3.26990	3.22921	3.18900	3.14925	3.10996	.422790
−52	3.07113	3.03275	2.99481	2.95731	2.92025	2.88362	2.84742	2.81165	2.77628	2.74134	.376464

Deg	0.0	0.1	0.2	0.3	0.4	0.5	0.6	0.7	0.8	0.9	Derivative

	Pa	Pa	Pa	Pa	Pa	Pa	Pa	Pa	Pa	Pa	Pa/K
C	MPa	MPa	MPa	MPa	MPa	MPa	MPa	MPa	MPa	MPa	MPa/K

−53	2.70680	2.67266		2.60559	2.57265	2.54009	2.50791	2.47611	2.44469	2.41364	.334847
−54 °	2.38296	2.35263		2.29306	2.26381	2.23490	2.20633	2.17810	2.15021	2.12265	.297501
⟜55	2.09542	2.06852		2.01567	1.98972	1.96408	1.93874	1.91371	1.88898	1.86455	.264024
−56	1.84042	1.81657		1.76974	1.74674	1.72403	1.70159	1.67942	1.65752	1.63589	.234047
−57	1.61452	1.59340		1.55195	1.53160	1.51150	1.49165	1.47204	1.45266	1.43353	.207235
−58	1.41463	1.39596		1.35931	1.34133	1.32356	1.30602	1.28869	1.27157	1.25467	.183280
−59	1.23797	1.22149		1.18912	1.17324	1.15756	1.14207	1.12678	1.11167	1.09676	.161902
−60	1.08203	1.06749		1.03894	1.02494	1.01111	.997462	.983980	.970668	.957524	.142846
−61	944.545	931.731	919.079	906.587	894.253	382.076	870.053	858.183	846.465	834.895	125.879
−62	823.473	812.196	801.064	790.074	779.225	768.514	757.941	747.504	737.201	727.030	110.790
−63	716.990	707.079	697.297	687.640	678.109	668.700	659.414	650.248	641.200	632.270	97.3887
−64	623.457	614.758	606.172	597.698	589.335	581.081	572.935	564.895	556.961	549.131	85.4990
−65	541.403	533.778	526.252	518.826	511.497	504.265	497.128	490.086	483.137	476.280	74.9642
−66	469.514	462.838	456.250	449.750	443.337	437.009	430.765	424.605	418.527	412.530	65.6416
−67	406.613	400.776	395.017	389.335	383.730	378.200	372.745	367.363	362.054	356.817	57.4022
−68	351.650	346.553	341.525	336.566	331.674	326.848	322.088	317.393	312.761	308.193	50.1295
−69	303.688	299.244	294.860	290.537	286.273	282.068	277.920	273.829	269.795	265.816	43.7185
−70	261.892	258.023	254.206	250.443	246.732	243.072	239.463	235.904	232.394	228.934	38.0746
−71	225.521	222.157	218.389	215.567	212.342	209.161	206.025	202.933	199.885	196.879	33.1128
−72	193.916	190.994	188.114	185.274	182.475	179.715	176.994	174.311	171.667	169.060	28.7565
−73	166.491	163.958	161.461	158.999	156.573	154.182	151.824	149.501	147.210	144.953	24.9372
−74	142.728	140.535	138.373	136.243	134.143	132.074	130.035	128.025	126.044	124.092	21.5935
−75	122.168	120.273	118.404	116.563	114.749	112.961	111.200	109.464	107.753	106.068	18.6702
−76	104.407	102.771	101.159	99.5705	98.0053	96.4631	94.9437	93.4468	91.9720	90.5190	16.1183
−77	89.0875	87.6772	86.2879	84.9192	83.5709	82.2427	80.9342	79.6453	78.3757	77.1250	13.8938
−78	75.8930	74.6795	73.4842	72.3069	71.1472	70.0050	68.8800	67.7720	66.6807	65.6059	11.9577
−79	64.5473	63.5047	62.4780	61.4668	60.4710	59.4904	58.5246	57.5736	56.6371	55.7149	10.2751
−80	54.8067	53.9125	53.0320	52.1649	51.3112	50.4706	49.6429	48.8280	48.0256	47.2356	8.81511
−81	46.4578	45.6921	44.9381	44.1959	43.4652	42.7458	42.0376	41.3405	40.6541	39.9785	7.55021
−82	39.3135	38.6588	38.0144	37.3800	36.7556	36.1410	35.5361	34.9407	34.3546	33.7778	6.45610
−83	33.2101	32.6514	32.1014	31.5602	31.0276	30.5034	29.9875	29.4799	28.9803	28.4886	5.51125
−84	28.0049	27.5288	27.0603	26.5994	26.1458	25.6995	25.2603	24.8282	24.4031	23.9848	4.69665
−85	23.5732	23.1683	22.7699	22.3780	21.9924	21.6131	21.2399	20.8728	20.5116	20.1563	3.99550
−86	19.8068	19.4630	19.1249	18.7922	18.4650	18.1432	17.8266	17.5152	17.2090	16.9077	3.39303
−87	16.6115	16.3201	16.0336	15.7517	15.4746	15.2020	14.9339	14.6703	14.4111	14.1562	2.87625
−88	13.9055	13.6590	13.4166	13.1783	12.9440	12.7135	12.4870	12.2642	12.0452	11.8299	2.43374
−89	11.6182	11.4100	11.2054	11.0042	10.8065	10.6120	10.4209	10.2330	10.0483	9.86680	2.05550
−90	9.68833	9.51290	9.34047	9.17098	9.00439	8.84064	8.67971	8.52153	8.36607	8.21329	1.73278
−91	8.06313	7.91556	7.77053	7.62801	7.48795	7.35031	7.21506	7.08216	6.95156	6.82323	1.45794
−92	6.69714	6.57324	6.45150	6.33189	6.21437	6.09890	5.98546	5.87401	5.76451	5.65694	1.22432
−93	5.55126	5.44745	5.34546	5.24528	5.14686	5.05019	4.95523	4.86195	4.77033	4.68034	1.02610
−94	4.59195	4.50513	4.41986	4.33612	4.25387	4.17310	4.09377	4.01586	3.93935	3.86422	0.858252
−95	3.79044	3.71799	3.64685	3.57699	3.50839	3.44103	3.37490	3.30997	3.24621	3.18361	.716394
−96	3.12216	3.06182	3.00258	2.94443	2.88734	2.83129	2.77627	2.72226	2.66924	2.61720	.596744
−97	2.56612	2.51597	2.46676	2.41845	2.37103	2.32450	2.27882	2.23400	2.19000	2.14683	.496029
−98	2.10445	2.06287	2.02207	1.98202	1.94273	1.90417	1.86634	1.82921	1.79279	1.75704	.411429
−99	1.72198	1.68757	1.65381	1.62069	1.58820	1.55632	1.52505	1.49437	1.46428	1.43476	.340513
−100	1.40580										

## Footnotes

1 Figures in brackets indicate the literature references at the end of this paper.

2 1 Pa = 1 N/m^2^ = 10^−5^ bar = 10^−2^ mb = 7.50062 × 10^−3^ mm Hg.

3 1 atmosphere = 101325 pascals.

4 The density was converted from g/ml to g/cm^3^ by using the factor 1 ml = 1.000028 cm^3^ [25].

5 The value given by Osborne was converted from international joules to absolute joules by the factor 1.000165 J = 1 i.j.

6 According to Eisenberg and Kauzman [44], who quote Shatenshtein et al. [45], the isotopic content of naturally occurring water depends on its origin. Within the limits of variation normally encountered the isotopes are H_2_^16^O, H_2_^17^O, H_2_^I8^O and HDO and the abundances are 99.73, 0.04. 0.20, and 0.03 percent, respectively. Combining these abundances with the relative atomic masses of the appropriate nuclides, recommended by the Commission on Atomic Weights [46], yielded the value 18.015277 grams for the molecular weight of naturally occurring water.
